# Expression of Concern: Bellomo et al. Effect of *Bifidobacterium bifidum* Supplementation in Newborns Born from Cesarean Section on Atopy, Respiratory Tract Infections, and Dyspeptic Syndromes: A Multicenter, Randomized, and Controlled Clinical Trial. *Microorganisms* 2024, *12*, 1093

**DOI:** 10.3390/microorganisms13061237

**Published:** 2025-05-28

**Authors:** Microorganisms Editorial Office

**Affiliations:** MDPI, St. Alban-Anlage 66, 4052 Basel, Switzerland

With this notice, the *Microorganisms* Editorial Office alerts the readers of concerns related to this article [1]. Following publication, concerns were raised to the Editorial Office regarding the degree in which this study adheres to MDPI’s policies on Research Involving Human Participants (https://www.mdpi.com/ethics#_bookmark9 accessed date: 18 May 2025). The Editor-in-Chief has decided to issue this expression of concern while an investigation is being conducted. The authors’ institution has been contacted to contribute to this investigation.

Once this process has been completed, the Editorial Office will provide an update on the situation and announce any post-publication modifications deemed necessary, as per MDPI’s Update to Published Papers policy (https://www.mdpi.com/ethics#_bookmark30 accessed date: 18 May 2025).
